# Comparison of publication trends in dermatology among Japan, South Korea and Mainland China

**DOI:** 10.1186/1471-5945-14-1

**Published:** 2014-01-09

**Authors:** Huibin Man, Shujun Xin, Weiping Bi, Chengzhi Lv, Theodora M Mauro, Peter M Elias, Mao-Qiang Man

**Affiliations:** 1 Wendeng Central Hospital, Shandong, P.R. China; 2 The Center for Skin Physiology Research, Dalian Skin Disease Hospital, Liaoning 116021, P.R. China; 3 Dermatology Service, Veterans Affairs Medical Center, San Francisco, CA, USA; 4 Department of Dermatology, University of California, 4150 Clement Street, San Francisco, CA 94121, USA

**Keywords:** Publication, Dermatology, Gross domestic product, China, Japan, South Korea

## Abstract

**Background:**

We previously showed that the number of publications in dermatology is increasing year by year, and positively correlates with improved economic conditions in mainland China, a still developing Asian country. However, the characteristics of publications in dermatology departments in more developed Asian countries such as Japan and South Korea are unknown.

**Methods:**

In the present study, publications from 2003 through 2012 in dermatology in Japan, South Korea and mainland China were characterized. All data were obtained from http://www.pubmed.com.

**Results:**

Dermatology departments in Japan published 4,094 papers, while mainland China and South Korea published 1528 and 1,758 articles, respectively. 48% of articles from dermatology in Japan were original research and 36% were case reports; The number of publications in Japan remained stable over time, but the overall impact factors per paper increased linearly over the last 10 year period (p < 0.05). In mainland China, 67% of articles from dermatology were original research, while 19% were case reports; The number of publications and their impact factors per paper increased markedly. In South Korea, 65% of articles from dermatology were original research and 20% were case reports. The impact factors per paper remained unchanged, despite of the fact that the number of publications increased over the last 10 year period (r^2^ = 0.6820, p = 0.0032). Only mainland China showed a positive correlation of the number of publications with gross domestic product per capita during this study period.

**Conclusions:**

These results suggest that the total number of publications in dermatology correlates with economic conditions only in developing country, but not in more developed countries in Asia. The extent of economic development could determine both the publication quantity and quality.

## Background

The total number of publications is considered as a key indicator of scientific productivity and is often used to evaluate the success of research [1-3]. The total number of publications tends to increase yearly in medical field. For instance, number of publications in otolaryngology research increased by over 80% from the year 1995 to 2000 [4]. The number of publications also increased in the dermatology field year by year [5,6]. There are many determinants, such as gross domestic product (GDP), number of medical school, number of dermatologists, language skills as well as population size that influence the quantity and quality of publications [1,7,8]. Funding, which is closely associated with economic conditions, is a key factor that impacts scientific productivity [9,10]. Although the number of publications strongly correlates with GDP [1,6], GDP does not always influence scientific productivity in certain nations. For example, from 2001 to 2010, the number of publications in anesthesia journals from the United States declined from 412 to 361, despite the fact that NIH funding for anesthesia research increased [11] and the GDP per capita increased from $35,912 to $46,612 during that period. Similarly, the number of publications from dermatology in Finland decreased slightly from 1989 to 2008 [8], although GDP per capita increased from $23,527 to $51,186. We have reported that the number of publications in dermatology from mainland China, a developing country, markedly increased over the last 10 years, strongly correlating with GDP per capita. These results suggest that the correlation between the number of publications and GDP varies from country to country, and that the number of publications could remain relative stable, or even decline in well-developed countries, despite increases in GDP and/or overall funding for research. In the present study, we compared the characteristics of publications over ten year period in dermatology in mainland China, South Korea and Japan which represent developing, developed and well-developed countries, respectively, in Asia.

## Methods

For publication searches, the internet address, http://www.ncbi.nlm.nih.gov/pubmed, was used to search articles in English from January 1, 2003 to December 31, 2012. The terms used to search each type of articles were listed in Table 1. Online Epub (ahead online electronically, but not yet printed) articles and papers published by authors not from each respective country were excluded. If publications were collaborated among countries, these papers were considered from all collaborating countries as long as the country names were listed in authors’ affiliations. Meeting abstracts, announcements or papers without author list were not included. Papers identified as case report with review were considered as review articles. Since English is thought to be the universal scientific language [4], only papers published in English were included. Since China was used for the search term for publications from dermatology in mainland China, articles from dermatology inTaiwan, Hongkong and Macau were excluded from the search results. Because the number of published papers is usually proportional to data for gross domestic product per capita (GDPPC), GDPPC from 2002 to 2011 (since usually prior year fund supports current year production) also were obtained from http://data.worldbank.org/indicator/NY.GDP.PCAP.CD. (Obtained on April 5, 2013).

**Table 1:** Search terms used in the study

**Types of articles searched**	**Search terms**
All types of articles	Dermatology, Japan or China or Korea
Review	Review, Dermatology, Japan or China or Korea
Case report	Case report, Dermatology, Japan or China or Korea
Clinical trial	Clinical trial , dermatology, Japan or China or Korea
Meta-analysis and letter	Meta-analysis, letter, Dermatology, Japan or China or Korea
Randomized controlled trial	Randomized controlled trial, dermatology, Japan or China or Korea

There were some limitations in this study. For example, if either respective nation’s name and/or dermatology were not listed as the authors’ affiliations in papers, they would not be disclosed by the search terms in the present study.

### Statistics

GraphPad Prism 4 software (San Diego, CA, USA) was used for all statistical analyses. Two-tailed nonparametric correlation and linear regression were used to determine significance.

## Results

### The number of annual publications increased linearly in Mainland China and South Korea, but not in Japan from 2003 to 2012

Dermatology in Japan published 4094 articles in 395 journals at an average of 10.37 articles per journal; 48% of papers were original research and 36% were case report (Table 2). Dermatology in mainland China published 1528 articles in 306 journals at an average of 4.99 articles per journal; 67% of papers were original research and 19% were case report (Table 2). A total of 1758 articles from dermatology in South Korea were published in 167 journals with an average of 10.53 articles per journal; 65% of papers were original research and 20% were case report (Table 2).

**Table 2:** Characteristics of overall publications in each country over the last 10 years

**Country**	**Number of publications (% of total publications)**						
	**Original research**	**Case report**	**Review**	**Clinical trial**	**Randomized controlled trial**	**Letter**	**Total**
**Japan**	1978 (49.31%)	1468 (35.86%)	440 (10.75%)	143 (3.49%)	47 (1.15%)	18 (0.44%)	4094 (100%)
**South Korea**	1142 (64.96%)	351 (19.97%)	71 (4.04%)	129 (7.34%)	64 (3.64%)	1 (0.06%)	1758 (100%)
**Mainland China**	1022 (66.88%)	290 (18.98%)	90 (5.89%)	81 (5.3%)	18 (2.42%)	8 (0.52%)	1528 (100%)

More than 50% of papers from South Korea and Japan were published in their favored10 journals (Table 3). Only 37% of articles from dermatology in mainland China were published in their favored 10 journals. In Japan, 23.55% of papers were published in Japanese journals, the Journal of Dermatology and the Journal of Dermatological Science. Likewise, the largest portion (30%) of articles from dermatology in South Korea was published in the Annals of Dermatology and the Journal of Korean Medical Science, both of which are Korean journals. In contrast, 9.2% of articles from dermatology in mainland China were published in the Chinese-owned journals, the Chinese Medical Journal and the Journal of Huazhong University of Science and Technology [Medical Sciences]. During this period, the Journal of Investigative Dermatology, the number one journal in dermatology field, published 209 articles from Japan, 34 from mainland China and 35 from South Korea.

**Table 3:** Leading journals for each country from 2003 to 2012

**Japan**			**South Korea**			**Mainland China**		
**Journals**	**Number of papers**	**% of total papers**	**Journals**	**Number of papers**	**% of total papers**	**Journals**	**Number of papers**	**% of total papers**
J Dermatol.	695	16.98	Ann Dermatol	463	26.34	Chin Med J (Engl).	82	5.37
J Dermatol Sci.	269	6.57	Dermatol Surg.	131	7.47	Int J Dermatol.	82	5.37
Br J Dermatol	233	5.69	J Dermatol.	121	6.90	Clin Exp Dermatol	79	5.17
J Invest Dermatol	209	5.11	Int J Dermatol	74	4.22	Arch Dermatol Res.	60	3.93
Clin Exp Dermatol.	174	4.25	Br J Dermatol.	72	4.10	Br J Dermatol.	60	3.93
Eur J Dermatol.	148	3.62	J Korean Med Sci.	66	3.75	J Huazhong Univ Sci Technolog Med Sci.	59	3.86
Dermatology.	142	3.47	Clin Exp Dermatol	65	3.71	J Eur Acad Dermatol Venereol.	43	2.81
Int J Dermatol.	126	3.08	Exp Dermatol	46	2.62	Mycopathologia.	40	2.62
Arch Dermatol Res	109	2.66	J Am Acad Dermatol.	45	2.57	J Invest Dermatol.	34	2.23
J Am Acad Dermatol.	107	2.61	J Dermatol Sci.	39	2.22	Eur J Dermatol.	33	2.16
Total	2212	54.03	Total	1122	63.83	Total	572	37.43

**Figure 1:**
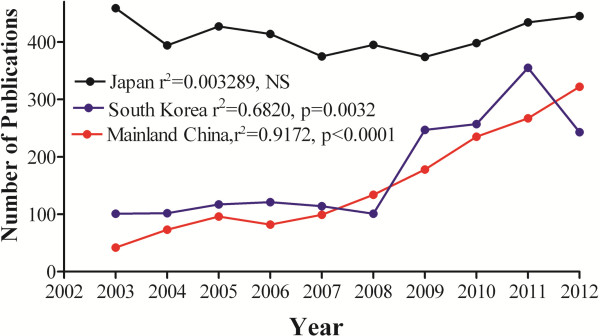
**Changes in the number of publications from 2003 to 2012.** Publication data were collected as described in materials and methods section. Linear regression was used to analyze the significance. Part of data (2003–2011) for mainland China was reported earlier [6].

The number of publications both in mainland China and South Korea rose linearly over the last 10 years, while the number of annual publications in Japan was similar during that period (Figure 1). Linear regression analysis showed that slopes for Japan, mainland China and South Korea were 1.05515, 29.67 and 24.93, respectively. Taken together with values of Y intercept for each country (1519 for Japan, -49880 for South Korea, and -59420 for mainland China), it was predicted that by middle of 2016 the number of publications in dermatology in mainland China would be comparable to that in Japan and the number of publications in dermatology in South Korea could catch Japan by early 2017.

### Changes in the quality of publications from 2003 to 2012

**Figure 2:**
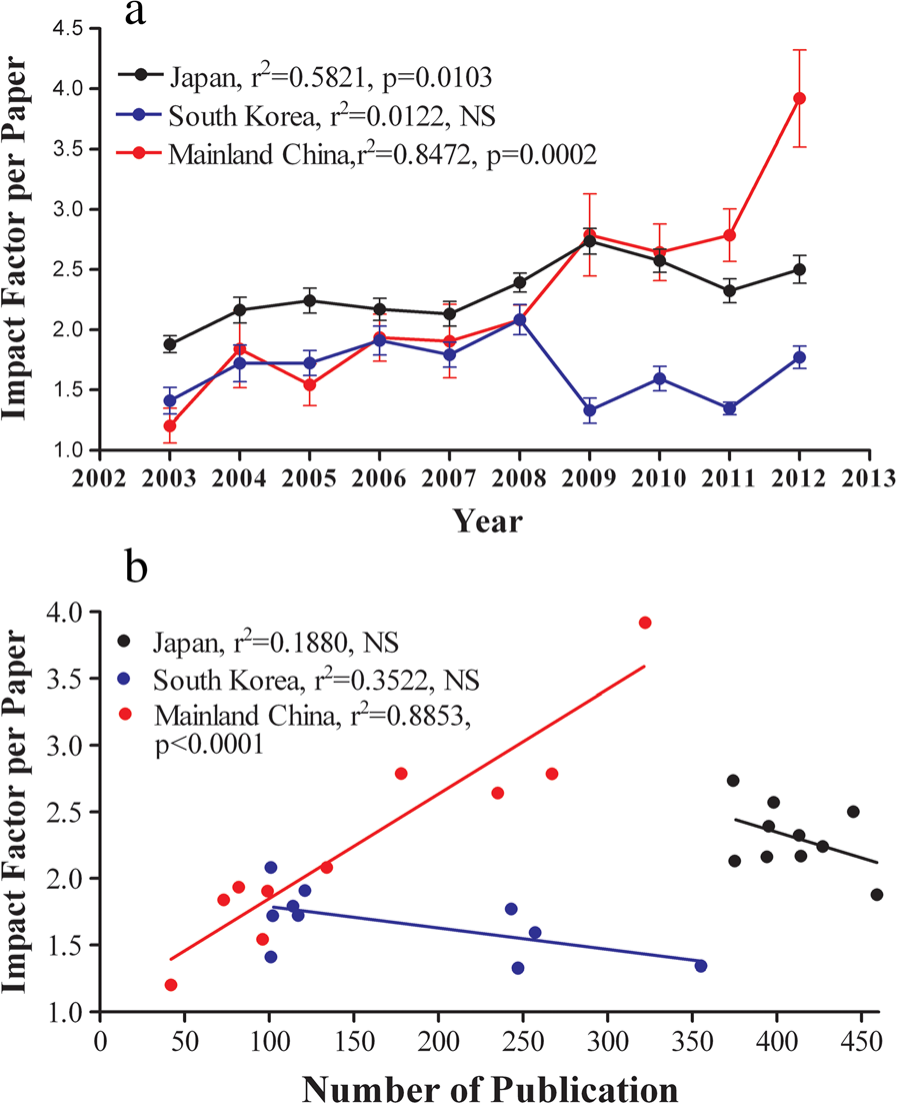
**The changes of average impact factor per paper and their relationship to number of publications.** Publication data were collected as described in materials and methods section. The impact factor per paper was calculated by dividing total impact factor by total number of published papers in each year. Linear regression was used to analyze the significant changes of impact factor per paper over the study period (Figure 2**a**). For correlation of impact factor per paper with the number of publication, Two-tailed Pearson test was used to determine significance (Figure 2**b**). Part of data (2003–2011) for mainland China in 3a was reported earlier [6].

Journal impact factors are often used to measure the quality of published research although only a small portion (25-30%) of publications in a journal can largely determine the impact factor for that journal [12-15]. We used the journals’ impact factors to determine changes in the quality of publications in Japan and South Korea between 2003 and 2012. Since the average impact factors rose steadily from 2002 to 2011 in a developing country, mainland China [6], we next assessed whether the quality of publications in South Korea and Japan also improved over the last 10 years. For better comparison, data of impact factor per paper in dermatology in mainland China over this period were also added. As seen in Figure 2a, over the whole 10 year period the average impact factor per paper in Japan and mainland China increased significantly, while in South Korea the average impact factors per paper did not change significantly. It is worth noting that the average impact factor per paper in both South Korea and Japan had tended to decline in the last 3 years. Since product quality can correlate negatively with quantity, we next correlated the average impact factor per paper with the number of papers. Indeed, the average impact factor per paper correlated negatively but weakly with the number of publications both in Japan and South Korea (Figure 2b). In contrast, in mainland China the average impact factor per paper correlated positively with the number of publications. These results suggest that remarkable improvement in publication quality occurs in developing country and publication quantity could trades for its quality in some countries.

### The impact of economic conditions on publications between 2003 and 2012

**Figure 3:**
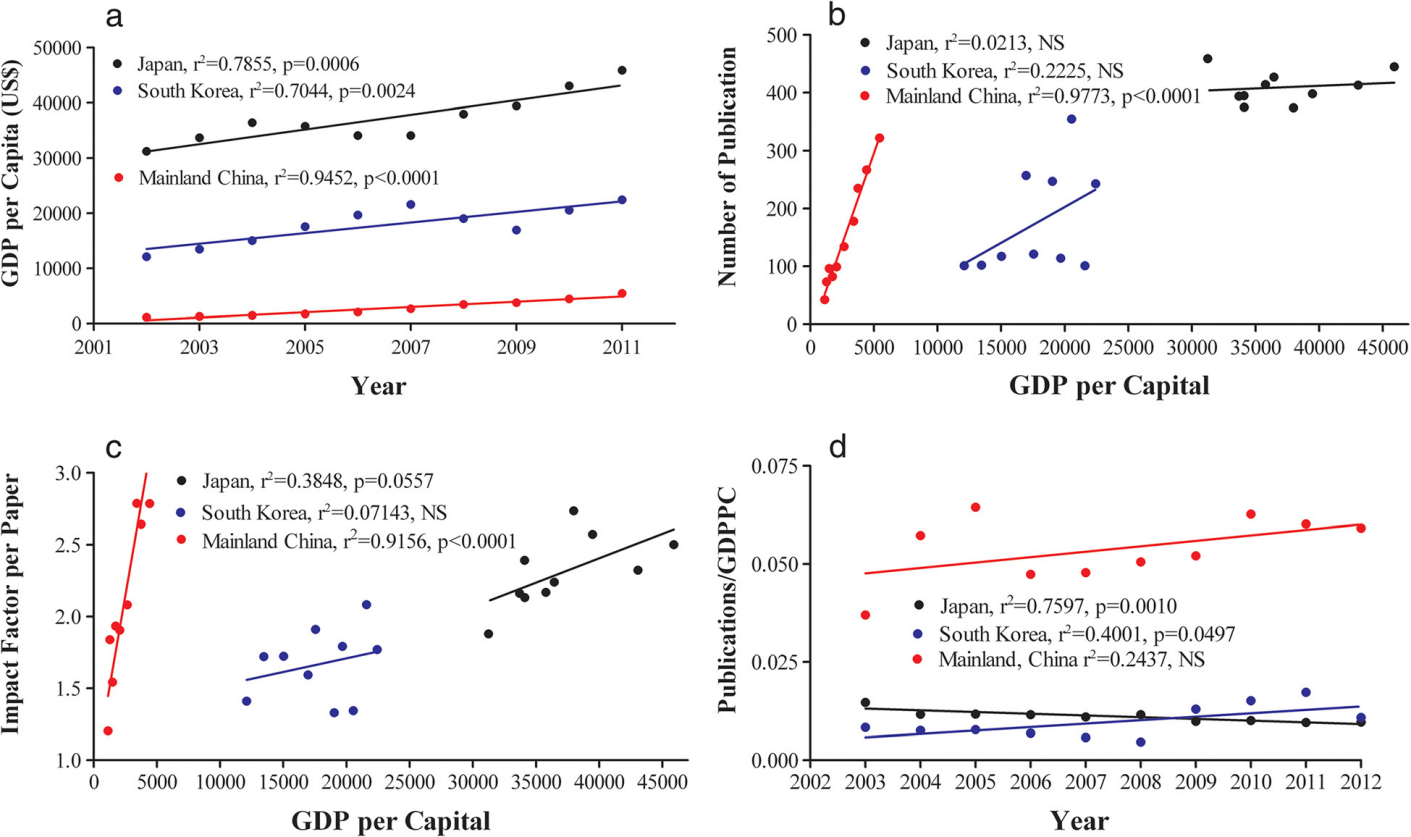
**The influence of GDPPC on publications over last 10 years.** Figure 3**a** exhibits the GDPPC in each year from 2003 to 2012. Linear regression was used to analyze the significance. Figure 3**b** and **c** display the correlation of number of publications and impact factor per paper with GDPPC, respectively. Two-tailed Pearson test was used to determine significance. Figure 3**d** exhibits the changes in the number of publications per GDPPC over the last 10 years. Linear regression was used to analyze the significance. Part of data (2003–2011) for mainland China in 4a and b was reported earlier [6].

Previous study showed that the number of publication correlated strongly with GDPPC in mainland China, a developing country in Asia [6]. We next determined whether GDPPC also influenced the quantity and quality of publications in South Korea and Japan, two developed countries in Asia. As seen in Figure 3a, GDPPCs in both Japan and South Korea increased linearly from 2002 to 2011. In contrast to the finding in developing country, mainland China [6], the number of publications did not correlate with the GDPPC in either Japan or South Korea (Figure 3b). The increased in GDPPC without increasing the number of publications could reflect an improvement in the quality of publications, as measured by impact factor per paper. Hence, we next correlated the average impact factor per paper with GDPPC. As seen in Figure 3c, the average impact factor per paper correlated positively and strongly with GDPPC in mainland China, weakly in Japan, but not in South Korea. The results indicate that economic conditions significantly impact both the quantity and quality of publications in dermatology in developing country, not developed countries in Asia.

To further reveal the impact of economic conditions on the number of publications in dermatology, changes in number of publication per GDPPC over the last 10 years were analyzed. The results showed that the number of publication per GDPPC in mainland China slightly increased over the last 10 years and was higher than that in Japan and South Korea (Figure 3d). A significant increase in the number of publication per GDPPC was observed in South Korea over this period. However, the number of publication per GDPPC in Japan declined significantly. These results demonstrate that mainland China, a developing country, yields higher number of publication per GDPPC in dermatology and further confirm that the impact of GDPPC on publications varies with nations.

## Discussion

In the present study, we compared the publication trends in dermatology in developing, developed and well-developed countries in Asia. Dermatology in Japan, the well-developed country, published papers more than the total of mainland China and South Korea over the last 10 years. It is reported that the number of publications in dermatology has been increasing yearly in both developing and developed countries [6,16] and the increase in the number of publications is associated with improvement of economic conditions [1,6,17]. In contrast, the present study revealed that the number of publications in dermatology in Japan remained unchanged from 2003 to 2012 despite the linearly increase of GDPPC. This result is consistent with the observation in other developed countries such as Sweden and Norway where the number of publications in dermatology was relatively stable from 1989 to 2008 [8]. But overall the number of publications in mainland China and South Korea increased over the last 10 years. These data suggest that well-developed countries can reach maximum research productivity in dermatology and GDPPC may no longer be the key determinant that influences the number of publications. In contrast to the number of publications, the impact factor per paper in Japan increased from 2003 to 2012. Although GDPPC did not correlated with the number of publications at all, the quality of publications was likely associated with GDPPC. Coupling with the findings that the number of publications slightly and reversely correlated with impact factor per paper, it suggests that dermatology researchers in Japan sacrifice publication quantity for quality. Therefore, the impact of GDPPC on dermatology research in Japan reflected in linearly increase of impact factor per paper from 2003 to 2012. Another potential factor refraining Japan from increasing the number of publications could be the stable population. It has been reported that the number of publications correlates with population [1]. The change in population was negligible in Japan from 2002 to 2011 (from 127445000 in 2002 to 127817277 in 2011, 0.29% increase; data were from http://data.worldbank.org/indicator/SP.POP.TOTL.). Thus, the changes of article number were marginal over the last 10 years. Regarding the decreased number of publications per GDPPC from 2003 to 2012, it could result from improved publication quality. Nevertheless, the present study indicates that the quality of publications in Japan improved from 2003 to 2012 although the quantity remains unchanged.

South Korea is a developed country in Asia. The number of publications dramatically increased in the last 10 years. There was a big increase in the number of publications in 2009 when a significant drop in impact factor per paper occurred. These changes could represent an example of trade-off between quantity and quality. The sudden increase of publication in 2009 could be also ascribed to the sharp addition of papers (over 40%) published in Annals of Dermatology, which was first indexed in PubMed in 2009. The overall increase of publications over the last 10 years could be attributed to both improved economic condition and increased population (from 47622000 in 2002 to 49779000 in 2011, 4.53% increase). GDPPC did not correlate with either the quantity or the quality of publications over the last 10 years in South Korea.

Both the quantity and the quality of publications in dermatology in mainland China were remarkably improved over the last 10 years. In addition to economic condition, manpower, language skills and promotion requirement [6], incentive award programs could also motivate researchers to publish more papers, especially in the journals with higher impact factor. Some institutions offer as much as 10,000 yuan RMB (about US$1,500) per impact factor for those papers published in high impact journals. Additionally, some institutions in mainland China use impact factors to determine the employment and promotion. Thus, incentive award programs and professional career requirement would definitely stimulate scientific research productivity, including dermatology research, in mainland China. In contrast to developed countries in Asia, GDPPC strongly correlate with both the quantity and the quality of publications in dermatology in mainland China.

Notably dermatology in both South Korea and Japan published significant portion of their papers in their top 10 favored journals. This is in agreement with those finding in mainland China [6]. However, the average number of papers per journal was much lower in mainland China (4.9 papers/journal) than in South Korea (10.53 papers/journal) and Japan (10.37 papers/journal). This may reflect that mainland China has a larger number of dermatologists and the broader range of research interests than South Korea and Japan.

In the present study, there are some limitations for using impact factor as a tool measuring the publication quality. First of all, although the impact factor usually represents the quality of a journal, approximately only 25% of papers largely determine the impact factor for a journal [12-15]. This means that not all papers published in journals with higher impact factor have higher impact. It is not uncommon that articles published in journals with lower impact factor have higher citations. Secondly, sometimes where to publish papers depends on the authors’ preference. Likewise, the acceptance of a manuscript could mainly rely on editor and reviewers’ preferences. For example, the present study revealed that authors in dermatology in South Korea prefer to publish their papers in the Annals of Dermatology and authors in dermatology in Japan prefer to publish their papers in the Journal of Dermatology. Finally, there are many factors such as the number of papers published by the journal, study field and type of articles that could affect journal impact factor [18]. Thus, impact factor may not truly reflect the impact or quality of every paper and extra caution should be taken when average impact factor per paper are compared between countries.

## Conclusions

The present study shows that the publication characteristics in dermatology over the last 10 years vary among developing, developed and well-developed country in Asia. The results also suggest that the quality of dermatology research could reversely correlate with quantity.

## Competing interests

All authors declare that they have no competing interests.

## Authors’ contributions

MH, XS, BW and LC collected and organized data from pubmed website; MMQ made figures and write draft; MTM and EPM helped design and critically reviewed manuscript. All authors read and approved the final manuscript.

## Pre-publication history

The pre-publication history for this paper can be accessed here:

http://www.biomedcentral.com/1471-5945/14/1/prepub
